# Negative Correlation Between ^18^F-RGD Uptake *via* PET and Tumoral PD-L1 Expression in Non-Small Cell Lung Cancer

**DOI:** 10.3389/fendo.2022.913631

**Published:** 2022-06-30

**Authors:** Leilei Wu, Jingru Liu, Shasha Wang, Menglin Bai, Min Wu, Zhenhua Gao, Jianing Li, Jinming Yu, Jie Liu, Xue Meng

**Affiliations:** ^1^ Department of Radiation Oncology, Shanghai Pulmonary Hospital, Tongji University School of Medicine, Shanghai, China; ^2^ Department of Radiation Oncology, Shandong Cancer Hospital and Institute, Shandong First Medical University and Shandong Academy of Medical Sciences, Jinan, China; ^3^ Department of Radiation Oncology, Shandong University Cancer Center, Jinan, China; ^4^ Department of Radiation Oncology and Shandong Provincial Key Laboratory of Radiation Oncology, Shandong Cancer Hospital and Institute, Shandong First Medical University and Shandong Academy of Medical Sciences, Jinan, China; ^5^ Research Unit of Radiation Oncology, Chinese Academy of Medical Sciences, Jinan, China

**Keywords:** 18 F-RGD PET, negative correlation, tumoral PD-L1 expression, SUVmax, non-small cell lung cancer

## Abstract

**Purpose:**

We investigated the correlation of ^18^F-AlF-NOTAPRGD2 (^18^F-RGD) uptake during positron emission tomography (PET) with tumoral programmed death-ligand 1 (PD-L1) expression and explored its potential in immune checkpoint inhibitor treatment.

**Methods:**

Forty-two mice were subcutaneously injected with CMT-167 lung carcinoma cells. A total of 30 mice with good growth tumor and good general condition were selected. ^18^F-RGD PET scanning was performed on days 0, 2, 4, 6, 9, and 11 with five mice per day. Immunohistochemistry (IHC) for PD-L1 was performed on each specimen obtained from tumors. Thirty patients with advanced non-small cell lung cancer (NSCLC) were scanned using ^18^F-RGD PET/CT, and Milliplex multifactor detection analyzed serum PD-1/PD-L1 expression of twenty-eight of them. Thirteen of them were analyzed immunohistochemically using core needle biopsy samples obtained from primary tumors.

**Results:**

Thirty mice were scanned by ^18^F-RGD PET/CT and analyzed for PD-L1 expression in tumor cells by IHC finally. Maximum standard uptake value (SUVmax) and mean SUV (SUVmean) were significantly lower in relatively-higher-PD-L1-expression tumors than in relatively-low-PD-L1-expression tumors (P < 0.05). In patients, the SUVmax was significantly negatively correlated with tumoral PD-L1 expression by IHC (P=0.014). SUVmean, peak SUV (SUVpeak), and gross tumor volume (GTV) were also negatively correlated with PD-L1, but without significance (P > 0.05). SUVmax, SUVmean, SUVpeak, and GTV were negatively correlated with serum PD-1 and PD-L1, but not significantly. According to the receiver operating characteristic curve analysis, significant correlations between SUVmax and tumoral PD-L1 expression in both mice and patients were present (P < 0.05).

**Conclusion:**

Higher ^18^F-RGD uptake is correlated with depressed PD-L1 expression in tumor cells, and SUVmax is the best parameter to display tumoral expression of PD-L1. ^18^F-RGD PET may be useful for reflecting the immune status of NSCLC.

## Introduction

Immune checkpoint inhibitors (ICIs), designed to target inhibitory immune checkpoint molecules, such as programmed death-ligand 1 (PD-L1) and its receptor, programmed death-1 (PD-1), have dramatically changed the treatment landscape in non-small cell lung cancer (NSCLC) (1). Currently, tumoral PD-L1 expression is the most widely used and recognized biomarker for evaluating the efficacy of ICI treatment (2). Nevertheless, immunohistochemistry (IHC) from invasive tumor sampling is essential to ascertain PD-L1 expression, and its widespread application has been extremely limited due to invasiveness, tumor heterogeneity, and unfeasible sampling. To overcome these obstacles, non-invasive diagnostic tools such as liquid biopsy and positron emission tomography/computed tomography (PET/CT) have been developed.

Angiogenesis is an important component of the tumor microenvironment (TME), which is often highly abnormal and heterogeneous, leading to impaired tissue perfusion and a hypoxic environment, resulting in tumor progression and treatment resistance (3, 4). High expression of integrin αVβ3 in activated endothelial cells, but not in resting vessel cells of normal regions, makes this protein a potential surrogate parameter for angiogenesis in tumors (5). Therefore, molecular imaging employing a cyclic arginine-glycine-aspartate (RGD) peptide has been developed to assess angiogenesis within the TME as a potential imaging approach, as it interacts specifically with integrin αVβ3 and exhibits a significant positive correlation with αVβ3 expression (6, 7). Additionally, ^18^F-AlF-NOTAPRGD2 (also referred to as ^18^F-RGD), a novel one-step labeled integrin αVβ3-targeting PET probe, has proven to be safe and effective (8). Several studies have demonstrated that ^18^F-RGD PET/CT can be used to clearly characterize tumor angiogenesis. Furthermore, the hypoxia-inducible factor (HIF) pathway is a master regulator of angiogenesis (9–11). Several studies have shown that HIF is related to PD-L1 expression (12–14). Therefore, we hypothesized that ^18^F-RGD PET/CT might be correlated with PD-L1 expression, through the function of HIF in the progress of angiogenesis. ^18^F-RGD PET/CT is likely to guide ICI treatment in NSCLC.

Currently, no studies have unveiled the relation between ^18^F-RGD uptake and PD-L1 expression. In this study, we aimed to investigate whether ^18^F-RGD uptake is associated with tumoral PD-L1 expression, and whether ^18^F-RGD PET/CT imaging can be used to guide ICI treatment in NSCLC.

## Materials and Methods

Our research included two parts: experiments on CMT-167 lung carcinoma-bearing mouse models and prospective research on clinical patients. We chose CMT-167 lung carcinoma cells because of their high immunogenicity.

### Experiments on CMT-167 Lung Carcinoma-Bearing Mouse Models

#### CMT-167 Lung Carcinoma-Bearing Mice Models

Female C57BL/6 mice (n=42, age, 6-8 weeks, weight, 18–20 g) were purchased from Beijing Hua Fukang pathogen−free animal breeding facility (approval no. SCXK (Jing) 2009−0008). A total of 1 × 10^6^ CMT-167 lung carcinoma cells were subcutaneously injected into the axillae of these mice to generate tumors. The tumor size and body weight of mice were measured every two days, and the tumor volume was calculated using the following formula: Tumor volume = (length × width^2^)/2. The animal rooms provided a constant temperature of 26°C, a relative humidity of 50−60% and natural light, with a 12/12-h light/dark cycle. The mice were fed a laboratory animal diet and sterile water ad libitum. A total of 30 mice with good growth tumor and good general condition were selected. We set the date as day 0 when the tumor reached 100 mm^3^. Finally, five mice were prepared for ^18^F-RGD PET/CT scanning on day 0, 2, 4, 6, 9, and 11, respectively. Effectively scanned mice were then sacrificed to isolate the tumors. Tumors were sectioned for IHC. This study was approved by the Ethical Committee of the Shandong Cancer Hospital and Institute (No. SDTHEC20130326).

#### ^18^F-RGD PET/CT Scanning

A lyophilized kit for labeling the PRGD2 peptide purchased from the Jiangsu Institute of Nuclear Medicine was employed according to a previously published study (15). The radiochemical purity of ^18^F−RGD exceeded 95%, and its specific radioactivity exceeded 37 GBq (1,000 mCi)/µmol. All micro-PET images were obtained with an Inveon PET scanner (Siemens Preclinical Solutions, LLC, Knoxville, TN, USA) using ^18^F−RGD. With the assistance of the positioning laser from the Inveon system, each tumor-bearing mouse was placed with its tumor located in the center of the field of view to achieve the highest imaging sensitivity. ^18^F-RGD PET scans were performed 60 min after tail-vein injection of ^18^F−RGD (2.4−3.5 MBq) under isoflurane anesthesia with 1.5% isoflurane in 100% oxygen at a flow rate of 1.5 L/min. The PET scans were acquired on a combined PET/CT scanner for 5 min per field of view. The PET images were reconstructed and analyzed using the OSEM−3D IAM software program (IS_v1.4.3 SP1; Siemens Preclinical Solutions, LLC).

#### Imaging Data Analysis

Two experienced nuclear medicine physicians examined all images using a double-blinded approach and aimed to reach a consensus. If no consensus was reached, a third chief physician participated in the examinations. The process of this detailed analysis was the same as that described in a previously published study (16).

### Prospective Research on Clinical Patients

#### Inclusion Criteria

All patients were prospectively enrolled in this study and met all the following criteria (1): aged 18 years or older with a Karnofsky Performance Score (KPS) ≥70 (2); histologically confirmed NSCLC; and (3) diagnosed with objectively measured lesions. Patients with autoimmune diseases, active infections, or severe heart and lung dysfunction were excluded. Our investigation of these candidates was performed in accordance with the Declaration of Helsinki (as revised in 2013) and approved by the Ethical Committee of Shandong Cancer Hospital and Institute (No. SDTHEC20130326). Signed formal consent was required for all the candidates prior to participation.

#### ^18^F-RGD PET/CT Scanning

A baseline ^18^F-RGD PET/CT scan was performed for each patient enrolled at the Shandong Cancer Hospital and Institute. The uptake values of the primary lesions were analyzed. The lyophilized kit for labeling the PRGD2 peptide was the same as that used for the mice. Fasting or specific CT contrast agents were not used in this study. The radiochemical ^18^F-RGD purity exceeded 95%, and the specific radioactivity exceeded 37 GBq (1,000 mCi)/μmol. The PET scans were acquired on a combined PET/CT scanner (GEMINITF Big Bore; Philips Healthcare). PET scans were performed 60 min after intravenous injection of ^18^F-RGD, at approximately 219.24 ± 25.7 MBq. Patients were required to remain calm and sustain slow breathing during image acquisition. The PET scans were acquired from the head to the thigh (with patients’ supine) and on a combined PET/CT scanner, with a duration of 5 min per field of view. The axial sampling thickness was 4.25 mm per slice after intravenous administration of ^18^F-RGD. A low-dose multi-slice helical CT was performed for anatomic imaging, localization purposes, and attenuation correction. Attenuation-corrected PET images, CT images, and fused PET/CT images were presented as coronal, sagittal, and transaxial slices, respectively, and were viewed using a Xeleris workstation (GE Healthcare).

#### Imaging Data Analysis

PET/CT images were analyzed by two qualified and experienced nuclear medicine physicians using a double−blinded approach without knowledge of the patients’ history. The analysis was assisted with the MIM software package (MIM, 6.1.0, Ohio, USA). If no consensus was reached, a third chief physician participated in the analyses. Regions of interest (ROIs) of lesions were drawn based on anatomical structure according to CT images and PET/CT fusion images for ROI accuracy, which were limited to the lesions for biopsy, and all were primary lesions. A vendor-provided automated contouring program was used to generate maximum standard uptake value (SUVmax) and mean SUV (SUVmean) of all tumors based on a 2.5 threshold. Peak SUV (SUVpeak) were acquired as the average SUV within a one cubic centimeter sphere, surrounding the voxel with the SUVmax. Gross tumor volume (GTV) was measured through attenuation-corrected ^18^F-RGD PET images using an SUV-based automated contouring program with an iso-counter threshold method based on 41% of the SUVmax, defined as the total volume of all tumors in the body in milliliters.

### IHC Staining

For mice, tumor sections and commercial antibodies (Abcam, Shanghai, China, ab130039) were used for IHC staining. For patients, tumor tissue samples obtained via core needle biopsy samples obtained from primary lesions were used for IHC staining. Rabbit monoclonal antibodies against PD-L1 (28-8, Abcam, Shanghai, China, AB205921) and PD-1 (EPR4877 (2), Abcam, Shanghai, China, ab137132) were used for IHC analysis. The reaction was visualized using SignalStain Boost IHC Detection Reagent (HRP, Rabbit). Tumor expression of PD-1 or PD-L1 was considered positive when membrane staining was observed. The following semiquantitative scoring method (6-step scoring system, ‘Cologne Score’) was used for PD-1 and PD-L1: 0 (<1%), 1 (≥1% and <5%), 2 (≥5% and <10%), 3 (≥10% and <25%), 4 (≥25% and <50%), 5 (≥50% and <75%) (17, 18). Tumors with a score ≥2 were graded as relatively high expression, and tumors with a score ≤1 were graded as having relatively low expression (a cut-off value of 5%). An Eclipse Ci-L photographic microscope (Nikon, Japan) and Media Cybemetics software (USA) were used to analyze tumoral expression of PD-1 and PD-L1. Two experienced pathologists were consulted to ensure the accuracy of semi-quantitative scoring approach used.

### Statistical Analysis

Statistical analyses were performed using SPSS software (version 26.0; SPSS Inc., Chicago, IL, USA). The parameters of ^18^F-RGD PET/CT were regarded as continuous variables, and tumoral expression of PD-1 and PD-L1 was classified as a variable. Correlations between imaging parameters and tumoral expression of PD-1 and PD-L1 were determined by Spearman or Pearson correlation analysis, depending on whether the parameters were normally distributed. Correlations between PET parameters and serum PD-1/PD-L1 (sPD-1/PD-L1) were evaluated using the Pearson correlation analysis. Receiver operating characteristic curve (ROC curve) and area under the ROC curve (AUC) analyses were used to describe the recognition accuracy of different parameters. Differences were considered statistically significant at p < 0.05.

## Results

### Experiments on CMT-167 Lung Carcinoma-Bearing Mouse Models

#### Correlations Between ^18^F-RGD PET/CT Imaging Parameters and Tumoral PD-L1 Expression in Mice

**Figure 1:**
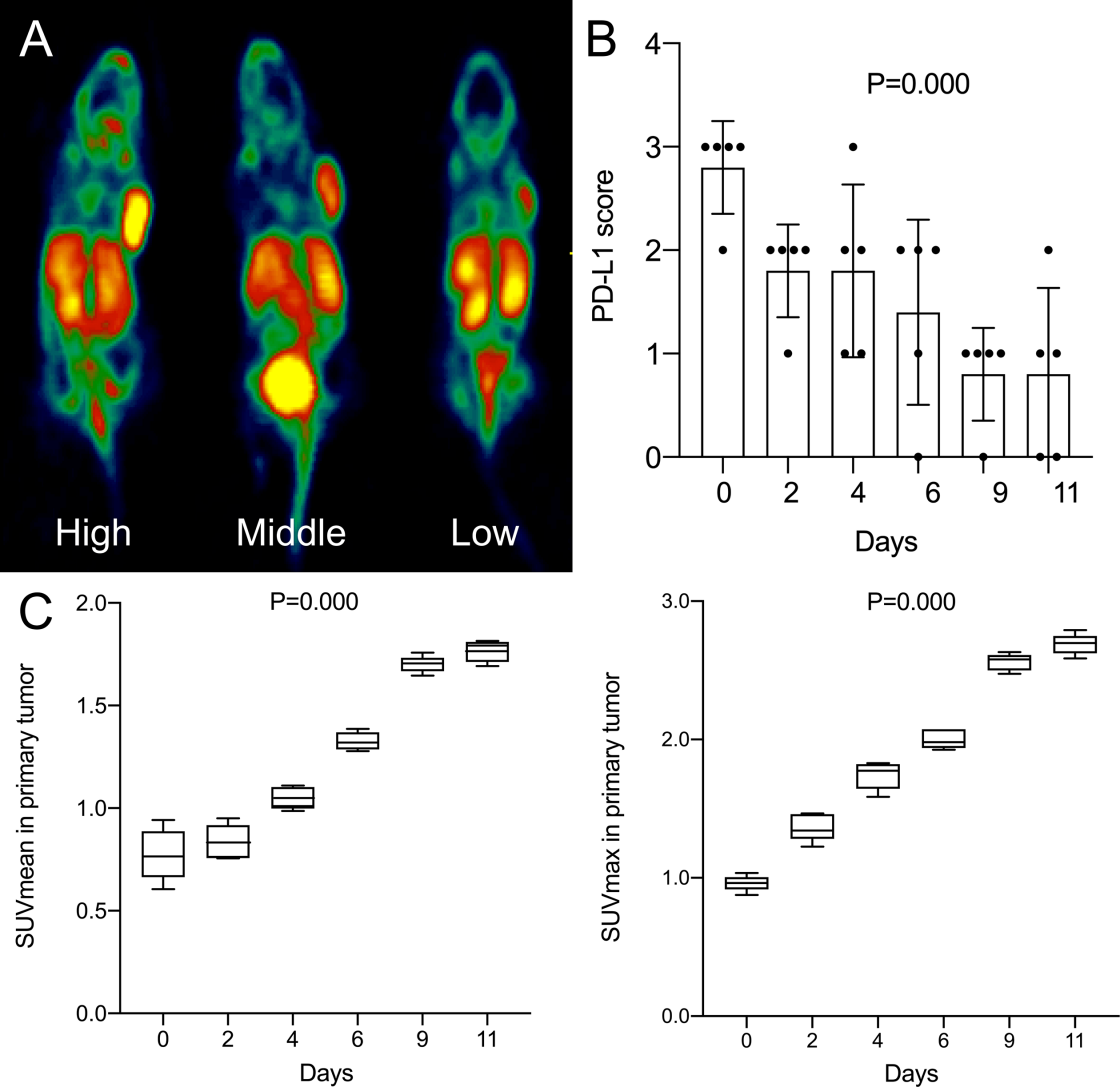
**(A)** High, middle and low uptake of tumor in mice by ^18^F-RGD PET/CT imaging; **(B)** Changes of tumoral PD-L1 expression score (6-step scoring system, ‘Cologne Score’) of mice with days; **(C)** Changes of SUVmean and SUVmax of mice with days. P < 0.05 is considered statistically significant.

**Figure 2:**
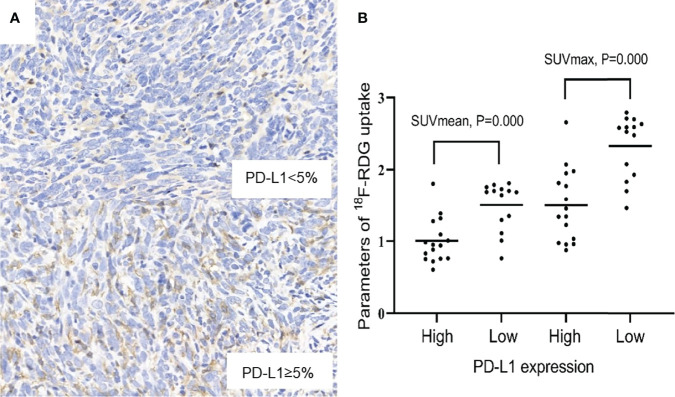
**(A)** Relatively low and high expression of PD-L1 (×400). **(B)** Scatter diagrams of correlations between SUVmax, SUVmean and PD-L1 expression. P < 0.05 is considered statistically significant.

As the days progressed, tumors gradually grew in size. SUVmean and SUVmax increased gradually, and there was a downward trend of tumoral PD-L1 expression over time (P=0.000; **Figure 1**). Specimens from mice were categorized into two groups (relatively low and high expression) according to the results of IHC analysis of tumoral PD-L1 expression. Both SUVmean (Pearson’s r = -0.623, P=0.000) and SUVmax (Pearson’s r = -0.667, P=0.000) were significantly lower in higher-PD-L1-expression tumors, in comparison with relatively-low-PD-L1-expression (**Figure 2**) (**Table 1**). When tumoral PD-L1 expression were classified into 6 scores, the highest score was 3 (17%), and both SUVmean (Pearson’s r = -0.682, P=0.000) and SUVmax (Pearson’s r = -0.726, P=0.000) were still significantly lower in higher-PD-L1-expression tumors (**Table 1**).

**Table 1:** Correlations between ^18^F-RGD PET/CT imaging parameters and tumoral PD-L1 expression in mice.

Variables		SUVmean		SUVmax	
		Pearson’s r	P	Pearson’s r	P
Tumoral PD-L1 expression	Dichotomy	-0.623	0.000	-0.667	0.000
	Six scoring categories	-0.682	0.000	-0.726	0.000

The dichotomy used a cut-off value of 5%. p < 0.05 is considered statistically significant.

### Prospective Research on Clinical Patients

#### Characteristics of Patients

Thirty patients received valid ^18^F-RGD PET/CT scanning before any anti-angiogenesis therapy or ICI treatment between December 2018 to March 2020, and 13 of these patients were analyzed immunohistochemically by core needle biopsy samples obtained from primary tumors. Of the 13 patients, eight (61.5%) were men, nine (69.2%) were older than 60 years, eight (61.5%) had a smoking history, and the tumor stage of nine (69.2%) participants had an IVA/B stage. KPS of one patient was 70 and six patients were evaluated as 80. Only one patient (7.7%) had squamous cell carcinoma, and three (23.1%) were poorly differentiated. The mean values and ranges of SUVmax, SUVmean, SUVpeak and GTV were 5.05 (3.00-12.00), 2.90 (1.00-4.00), 3.41 (2.00-6.00) and 25.14 (0.44-141.29), respectively. The percentages associated with PD-1 and PD-L1 positive expression were 7.7% and 53.8%, respectively. No significant differences in sex, age, smoking history, KPS, tumor stage, histopathologic subtype, differentiated degree, or epidermal growth factor receptor *(EGFR)* mutation were found between the relatively-high-PD-L1-expression tumors and relatively-low-PD-L1-expression tumors. The detailed characteristics of the 30 patients are listed in **Table 2**.

**Table 2:** Thirteen out of the thirty patients were analyzed immunohistochemically by samples obtained from primary tumors by core needle biopsy and they are highlighted in bold.

No.	Gender	Age	Smoking history	KPS	Tumor stage	Histopathologic type	Differentiation	*EGFR* mutaion	Uptake of 18F-RGD PET/CT				PD-1(IHC)	PD-L1 (IHC)	Multifacterdetection
									SUVmax	SUVmean	SUVpeak	GTV			
**1**	**F**	**73**	**N**	**80**	**IVB**	**A**	**Middle**	**N**	**7.52**	**3.42**	**5.58**	**12.67**	**L**	**L**	**Y**
**2**	**M**	**68**	**Y**	**90**	**IVB**	**A**	**High**	**-**	**3.76**	**2.70**	**3.23**	**19.31**	**L**	**H**	**Y**
**3**	**M**	**69**	**Y**	**80**	**IIIC**	**S**	**Middle**	**N**	**7.01**	**3.57**	**5.61**	**43.97**	**L**	**H**	**Y**
**4**	**F**	**65**	**Y**	**90**	**IIIB**	**A**	**Middle**	**-**	**7.99**	**3.39**	**5.02**	**45.75**	**L**	**L**	**Y**
**5**	**M**	**69**	**N**	**80**	**IVB**	**A**	**High**	**N**	**12.92**	**8.59**	**3.39**	**394.97**	**L**	**L**	**Y**
**6**	**F**	**49**	**Y**	**90**	**IVA**	**A**	**High**	**+**	**11.10**	**3.79**	**4.39**	**67.30**	**L**	**H**	**Y**
**7**	**F**	**62**	**N**	**90**	**IVB**	**A**	**High**	**N**	**13.95**	**3.66**	**9.08**	**140.07**	**L**	**L**	**Y**
**8**	**M**	**60**	**N**	**80**	**IVB**	**A**	**Middle**	**-**	**3.93**	**2.86**	**2.98**	**2.32**	**H**	**H**	**Y**
**9**	**M**	**59**	**Y**	**90**	**IIIB**	**A**	**Low**	**-**	**2.61**	**1.32**	**2.05**	**45.95**	**L**	**H**	**Y**
**10**	**M**	**62**	**Y**	**80**	**IIIC**	**A**	**Low**	**+**	**3.84**	**2.86**	**3.00**	**8.20**	**L**	**L**	**Y**
**11**	**M**	**63**	**Y**	**80**	**IVB**	**A**	**High**	**-**	**12.44**	**3.37**	**3.99**	**141.29**	**L**	**L**	**N**
**12**	**F**	**52**	**N**	**90**	**IVB**	**A**	**Low**	**-**	**2.86**	**2.60**	**2.36**	**7.52**	**L**	**H**	**Y**
**13**	**M**	**68**	**Y**	**70**	**IVB**	**A**	**Middle**	**-**	**2.80**	**2.58**	**2.36**	**0.44**	**L**	**H**	**Y**
14	F	42	N	80	IIIC	A	High	N	5.29	3.34	4.24	17.50			Y
15	M	70	Y	90	IVA	A	High	–	5.34	3.04	4.40	65.59			Y
16	M	60	Y	70	IVA	A	High	+	6.40	2.91	4.34	278.43			Y
17	F	58	N	90	IVB	A	Low	–	7.63	4.01	6.69	148.38			Y
18	M	35	N	90	IVA	A	High	+	4.59	3.02	3.84	21.45			Y
19	M	52	Y	80	IVB	A	High	–	5.01	3.00	4.15	12.30			Y
20	M	54	Y	70	IVB	S	Low	N	5.38	3.19	4.71	67.74			Y
21	F	66	N	80	IVB	A	High	–	4.23	2.86	3.65	23.25			**N**
22	M	65	Y	80	IVB	A	Middle	N	4.60	2.98	3.78	38.78			Y
23	M	61	Y	80	IVA	A	High	+	4.21	2.98	3.64	30.29			Y
24	M	64	N	90	IVB	A	Middle	–	5.34	3.06	3.99	25.37			Y
25	F	56	N	80	IVA	A	High	+	4.21	2.91	3.17	2.82			Y
26	M	69	Y	80	IVB	A	Middle	–	6.28	3.1	5.38	75.49			Y
27	F	66	N	90	IVB	A	Low	–	9.78	3.42	6.27	47.23			Y
28	M	68	Y	80	IVB	A	Low	–	4.59	3.00	3.55	2.40			Y
29	M	31	N	90	IVB	A	High	–	6.83	3.08	5.40	23.29			Y
30	M	66	Y	80	IIIC	A	High	–	11.03	3.98	8.83	166.50			Y

Twenty-eight of these patients received Milliplex multifactor detection for sPD-1/PD-L1 expressed in peripheral blood. Thirteen out of the thirty patients were analyzed immunohistochemically by samples obtained from primary tumors by core needle biopsy and they are highlighted in bold. L, relatively low expression; H, relatively high expression; Y, yes.

#### Correlations Between ^18^F-RGD PET/CT Imaging Parameters and Tumoral PD-1/PD-L1 Expression in NSCLC Patients

**Figure 3:**
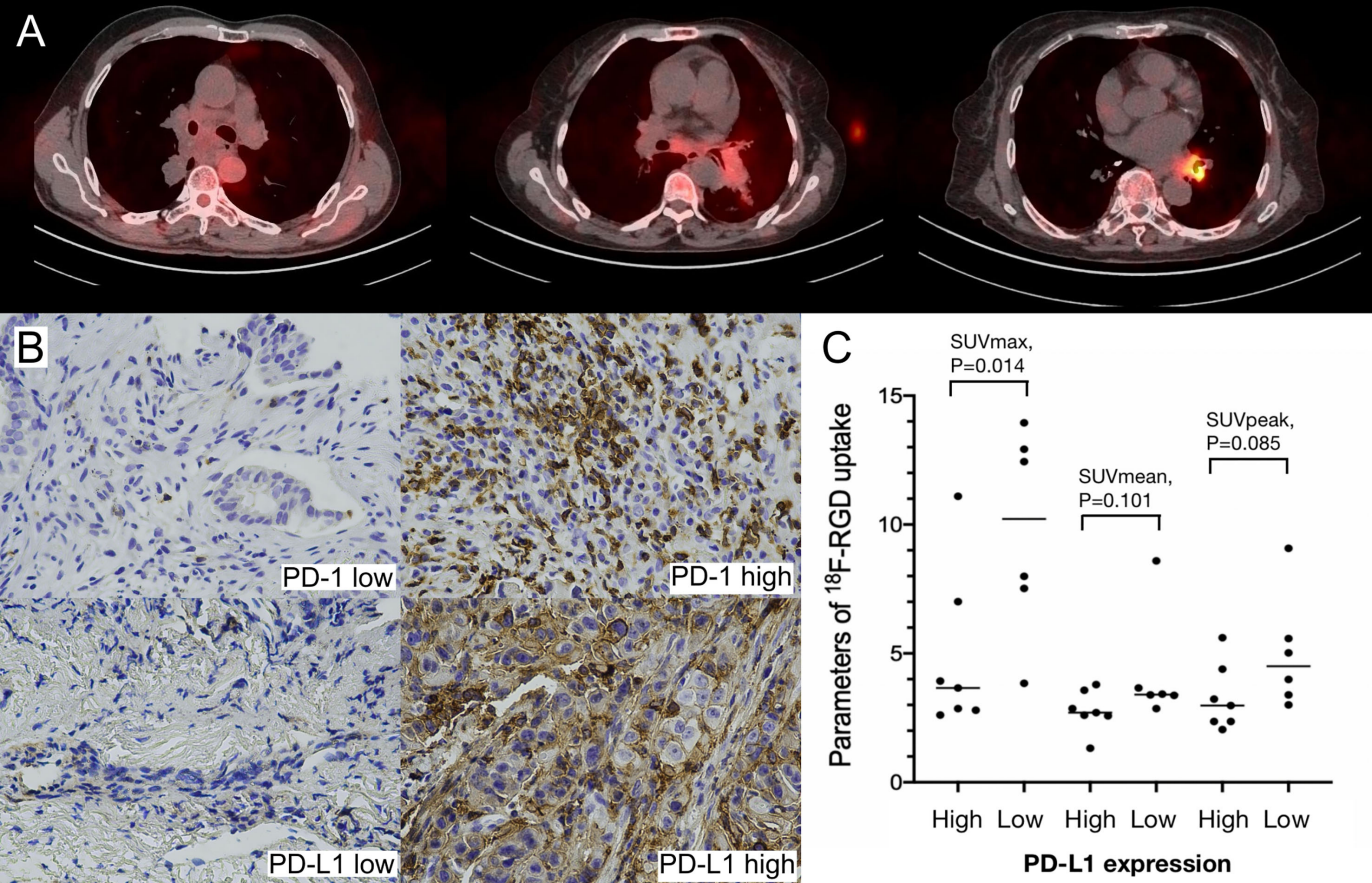
**(A)** Low, middle and high uptakes of ^18^F-RGD PET/CT scanning for NSCLC patients; **(B)** Relatively low and high expression of PD-1 and PD-L1 (×400) in NSCLC patients; **(C)** Scatter diagrams of correlations between SUVmax, SUVmean, SUVpeak and PD-L1 expression in NSCLC patients. P < 0.05 is considered statistically significant.

SUVmax, SUVmean, and GTV were positively correlated with greatest tumor diameter, but SUVpeak was not significantly correlated with greatest tumor diameter (**Table 3**). A statistically significant (Spearman’s r=-0.660, P=0.014) negative correlation between tumoral PD-L1 expression and ^18^F-RGD SUVmax was observed (**Table 3**). ^18^F-RGD SUVmean, SUVpeak, and GTV were slightly lower in tumor samples with higher PD-L1, although the differences were not statistically significant (P=0.101, P=0.085, and P=0.119, respectively). There was no significant relationship between SUVmax and tumoral PD-1 expression (Spearman’s r=-0.077, P=0.802). Tumoral expression of PD-L1 was also not significantly related to PD-1 expression (P=0.377). SUVmax was significantly positively correlated with SUVmean, SUVpeak, and GTV (P=0.000, P=0.002, and P=0.006, respectively). The ^18^F-RGD PET/CT scanning results are shown in **Figure 3A**, while representative specimens of NSCLC patient tissues showing tumoral PD-1 and PD-L1 expression (×400) are shown in **Figure 3B**. Finally, scatter diagrams showing correlations between SUVmax, SUVmean, SUVpeak and tumoral PD-L1 expression are shown in **Figure 3C**.

**Table 3:** Correlation with ^18^F-RGD uptake and greatest tumor diameter and tumoral PD-1/PD-L1 expression by IHC staining in patients.

Variables	SUVmax		SUVmean		SUVpeak		GTV	
	Spearman r	P	Spearman r	P	Spearman r	P	Spearman r	P
Greatest tumor diameter	0.663	0.013	0.689	0.009	0.253	0.405	0.674	0.011
Tumoral PD-1 expression	-0.077	0.802	-0.116	0.706	-0.232	0.446	-0.386	0.193
Tumoral PD-L1 expression	-0.660	0.014	-0.475	0.101	-0.496	0.085	-0.454	0.119

P < 0.05 is considered statistically significant.

#### Correlations Between ^18^F-RGD PET/CT Imaging Parameters and sPD-1/PD-L1

All parameters were evaluated as continuous variables. sPD-1 and sPD-L1 levels were significantly and positively correlated (P=0.000). SUVmax, SUVmean, SUVpeak, and GTV were negatively correlated with sPD-1 and sPD-L1, but not significantly. The correlations are presented in **Table 4**.

**Table 4:** Correlations Between Uptakes of ^18^F-RGD PET/CT and sPD-1/PD-L1 Expressed in Peripheral Blood of Twenty-Eight Patients.

Variables	SUVmax		SUVmean		SUVpeak		GTV	
	Pearson r	P	Pearson r	P	Pearson r	P	Pearson r	P
sPD-1	-0.078	0.693	-0.225	0.250	-0.040	0.841	-0.043	0.827
sPD-L1	-0.073	0.71	-0.205	0.295	-0.001	0.997	-0.016	0.935

P < 0.05 is considered statistically significant.

### ROC Curve Analysis

**Figure 4:**
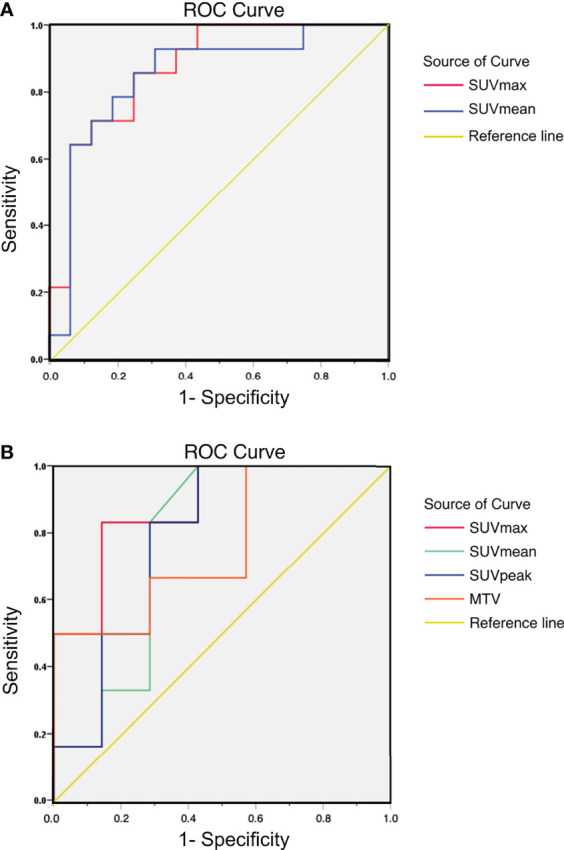
**(A)** ROC curve of ^18^F-RGD PET/CT parameters to reflect tumoral PD-L1 expression status (relatively high or low expression) in tumor-bearing mice: AUC of SUVmax and SUVmean were 0.871 (95% CI, 0.743-0.998) and 0.848 (95% CI, 0.699-0.997), respectively; **(B)** ROC curve of ^18^F-RGD PET/CT parameters to reflect tumoral PD-L1 expression status (relatively high or low expression) in NSCLC patients: AUC of SUVmax, SUVmean, SUVpeak and GTV were 0.881 (95% CI, 0.692-1.000), 0.774 (95% CI, 0.499-1.000), 0.786 (95% CI, 0.525-1.000) and 0.762 (95% CI, 0.490-1.000) respectively.

For tumor-bearing mice, there were significant correlations between SUVmax, SUVmean, and tumoral PD-L1 expression status (P=0.001 and P=0.001, respectively). The area under the curve (AUC) of SUVmax and SUVmean were 0.871 (95% confidence interval, 95%CI, 0.743-0.998) and 0.848 (95% CI, 0.699-0.997), respectively. For patients, significant correlations were observed between SUVmax and tumoral PD-L1 expression status (P=0.022), and the AUC of SUVmax was 0.881 (95% CI, 0.692-1.000). The correlations between SUVmean, SUVpeak, GTV, and tumoral PD-L1 expression were not significant (P=0.100, P=0.086, and P=0.116, respectively). The results of the ROC curve analysis are shown in **Figure 4** and **Table 5**.

**Table 5:** ROC curve of ^18^F-RGD PET/CT parameters to predict tumoral PD-L1 expression status (relatively high/low) in tumor-bearing mice and patients.

Parameters	ROC Curve Analysis for Mice		ROC Curve Analysis for Patients	
	AUC	P	AUC	P
SUVmax	0.871(0.743-0.998)	0.001	0.881 (0.692-1.000)	0.022
SUVmean	0.848 (0.699-0.997)	0.001	0.774 (0.499-1.000)	0.100
SUVpeak	–	–	0.786 (0.525-1.000)	0.086
GTV	–	–	0.762 (0.490-1.000)	0.116

P < 0.05 is considered statistically significant.

## Discussion

To our knowledge, this is the first study to provide evidence for the potential of ^18^F-RGD PET/CT imaging to indicate PD-L1 status in NSCLC. Furthermore, the results presented here suggest that ^18^F-RGD PET/CT may play a key role in determining optimal treatment strategies in patients with NSCLC. We found that uptake of ^18^F-RGD PET/CT was significantly negatively correlated with tumoral expression of PD-L1 in NSCLC, and SUVmax was the best parameter to reflect tumoral PD-L1 expression.

In recent years, the development of antibodies that target immune checkpoint proteins, including PD-1, PD-L1, and cytotoxic T lymphocyte-associated antigen-4, has led to a paradigm shift in cancer treatment and the US Food and Drug Administration approval of numerous therapeutics of this type, including for nivolumab, pembrolizumab, ipilimumab, and atezolizumab (17). However, the majority of patients fail to benefit from such treatments as they do not respond to ICIs, or quickly develop drug resistance. An essential and common factor associated with the suboptimal success of these drugs is the need for more suitable predictors of their therapeutic effect. The tumoral expression of PD-1 and its ligand PD-L1, as assessed by IHC, has been evaluated as a predictive biomarker of response to ICIs (2). However, due to the requirement for invasive biopsy, alternative noninvasive strategies that can indicate PD-1/PD-L1 expression in patients with malignant tumors, such as PET/CT and biochemical indexes of peripheral blood, would be of great value.

In our study, higher SUVs of ^18^F-RGD correlated with greatest tumor diameter in clinical patients. Similarly, in mouse models, as tumor size increased, ^18^F-RGD uptake increased, reflected as higher SUVmean and SUVmax. Such results revealed a significant positive correlation between the tumor dimension and RGD uptake values, which was concordant with the results of Li et al. (9). This trend confirmed the validity of these imaging parameters to reflect the true dimension of the tumors again and demonstrated the potential of RGD PET/CT to assess the TME. In terms of the mechanism of RGD PET to characterize tumor angiogenesis, highly expressive integrin αVβ3 in additional neovascularization with the growth of tumor manifests as elevated SUVs (5–9).

In CMT-167 lung carcinoma-bearing mice, SUVmax and SUVmean were significantly lower in relatively-higher-PD-L1-expression tumors, in comparison to relatively-low-PD-L1-expression tumors (P < 0.05). In patients, the SUVmax was also significantly negatively associated with PD-L1 expression by IHC (P=0.014). However, SUVmax, SUVmean, SUVpeak, and GTV were negatively correlated with sPD-1 and sPD-L1, but not significantly. Just like other tumoral markers in blood, sPD-1/PD-L1 can reflect tumor immune status and predict efficacy of ICIs to some extent, but the level of sPD-1/PD-L1 is affected by a variety of factors. It has been reported that the expression levels of serum PD-1/PD-L1 are not completely consistent with levels of PD-1/PD-L1 in tumor tissues (19, 20). There is a general agreement that expression of PD-L1 on tumor cells should be used to predict the therapeutic response to PD-1/PD-L1 inhibitors in NSCLC. Given the established positive association between ^18^F-RGD uptake and angiogenesis or integrin αVβ3, we speculate that angiogenesis may downregulate tumoral PD-L1 expression in NSCLC, corresponding to high ^18^F-RGD uptake. With increasing malignancy, hypoxia as a major TME factor mediates angiogenesis, regulated by HIF pathway (21, 22). The expression of pro-angiogenic genes such as vascular endothelial growth factor (VEGF) are regulated *via* the HIF pathway (21). Meanwhile, hypoxia also profoundly influences the immune status (22, 23). Thus, the possible correlation between ^18^F-RGD uptake and PD-L1 expression in cancer tissues may depend on the regulation of HIF pathway.

Several studies on the relationship between hypoxia or HIF and tumoral PD-L1 expression have yields conflicting results. Noman et al. reported that HIF-1α significantly increased the expression of PD-L1 in a panel of murine and human cell lines (12). However, only one (B16-F10 melanoma) of four murine cell lines studied showed an increase in the percentage of PD-L1 positive cells after culture in 0.1% oxygen. A study of the effect of hypoxia on PD-L1 expression in bladder cancer observed that hypoxia reduced PD-L1 expression and PD-L1 expression further decreased with increasing cell seeding density (24). It seems that the hypoxia-induced regulation of PD-L1 is tissue specific (12, 22, 24). E-cadherin is a tumor suppressor protein involved in cell-to-cell adhesion, and its suppression is a typical characteristic of epithelial-to-mesenchymal transition (EMT) induction (25, 26). Further studies revealed that HIF-1α activation upregulates PD-L1 expression by HIF-1α-induced EMT induction (27). Interestingly, PD-L1 expression has also been reported to be downregulated by EMT induction (28). A study on the diagnostic value of ^18^F-FDG-PET in predicting tumor immune status found that tumoral PD-L1 expression in oral squamous cell carcinoma (OSCC) patients is suppressed by the activation of the HIF-1α–EMT axis (29). Thus, how the HIF-EMT axis works may also determine the correlation between the uptake of ^18^F-RGD and PD-L1 expression in NSCLC. In other words, HIF-1α–EMT axis plays a crucial part in regulating the relationship between integrin αVβ3 and tumoral PD-L1 expression. Based on our results, we hypothesize that hypoxia produced during tumor growth further contributes to increased angiogenesis in the tumor microenvironment of NSCLC, resulting in robust expression of integrin αVβ3 and high RGD SUVs, and it downregulates the tumoral PD-L1 expression through the HIF-EMT axis simultaneously.

In recent years, molecular imaging, based on PET/CT, not only helps nuclear medicine physicians to diagnose diseases, but also plays an important role in predicting therapeutic effects. In addition to ^18^F-fluorodeoxyglucose (30), ^18^F-labeled anti-PD-L1 adnectin, ^89^Zr-labeled nivolumab (31), and ^99m^Tc-labeled anti-PD-L1 single-domain antibody (32), our study also provides clinical physicians with a new non-invasive measurements of PD-L1, which may be useful in guiding ICI treatment in NSCLC.

This study has some important limitations. First, the number of patients in the study was small, due to the infrequent use of ^18^F-RGD PET/CT in clinical practice. Second, we did not evaluate immune status using more detailed markers. Third, further research is warranted to investigate the specific correlation between angiogenesis and immune status.

## Conclusion

Higher expression of tumoral PD-L1 presented with lower ^18^F-RGD uptake, and SUVmax may be the best parameter to reflect the immune status of NSCLC. Metabolic imaging has the potential to become a useful complement in the assessment of the molecular profiles of NSCLC, and may be useful in guiding ICI treatment in NSCLC. Additional large, prospective clinical trials are required to confirm our results and to determine whether metabolic imaging could be useful, not only to infer the PD-1/PD-L1 status of patients but also to assist in making clinical decisions regarding whether the use of anti-PD-1/PD-L1 antibody therapy would be beneficial to the patient.

## Data Availability Statement

The raw data supporting the conclusions of this article will be made available by the authors, without undue reservation.

## Ethics Statement

The studies involving human participants were reviewed and approved by the Ethical Committee of Shandong Cancer Hospital and Institute (No. SDTHEC20130326). The patients/participants provided their written informed consent to participate in this study. The animal study was reviewed and approved by the Ethical Committee of Shandong Cancer Hospital and Institute (No. SDTHEC20130326). Written informed consent was obtained from the individual(s) for the publication of any potentially identifiable images or data included in this article.

## Author Contributions

LW was responsible for a part of experiments, data analysis and writing manuscript. JRL was responsible for a part of data analysis and writing manuscript. SW was responsible for a part of experiments. MB was responsible for a part of animal experiments. MW was responsible for a part of data analysis. ZG and JNL were responsible for selecting patient. JY provided improvement suggestions. JL was responsible for a part of experiments and provided improvement suggestions. XM provided financial support, improvement suggestions and was charge of manuscript correcting. All authors contributed to the article and approved the submitted version.

## Funding

JY has received grants from the Academic Promotion Program of Shandong First Medical University (2019ZL002), Research Unit of Radiation Oncology, Chinese Academy of Medical Sciences (2019RU071), the foundation of National Natural Science Foundation of China (81627901, 81972863 and 82030082), the foundation of Natural Science Foundation of Shandong (ZR201911040452). XM has received grants from National Natural Science Foundation of China (81972864 and 82172720), Science and Technology Support Plan for Youth Innovation Teams of Universities in Shandong Province (2019KJL001), Bethune·Translational Medicine Research Fundation for Tumor Radiotherapy (flzh202106).

## Conflict of Interest

The authors declare that the research was conducted in the absence of any commercial or financial relationships that could be construed as a potential conflict of interest.

The reviewer YC declared a shared affiliation with the authors JL, MB, MW, JY, and XM to the handling editor at the time of review.

## Publisher’s Note

All claims expressed in this article are solely those of the authors and do not necessarily represent those of their affiliated organizations, or those of the publisher, the editors and the reviewers. Any product that may be evaluated in this article, or claim that may be made by its manufacturer, is not guaranteed or endorsed by the publisher.

## References

[1] Mahoney KM Rennert PD Freeman GJ. Combination Cancer Immunotherapy and New Immunomodulatory Targets. Nat Rev Drug Discov (2015) 14(8):561–84. doi: 10.1038/nrd4591 26228759

[2] Gibney GT Weiner LM Atkins MB. Predictive Biomarkers for Checkpoint Inhibitor-Based Immunotherapy. Lancet Oncol (2016) 17(12):e542–e51. doi: 10.1016/S1470-2045(16)30406-5 PMC5702534 27924752

[3] Shweiki D Itin A Soffer D Keshet E. Vascular Endothelial Growth Factor Induced by Hypoxia may Mediate Hypoxia-Initiated Angiogenesis. Nature (1992) 359(6398):843–5. doi: 10.1038/359843a0 1279431

[4] Jain RK. Antiangiogenesis Strategies Revisited: From Starving Tumors to Alleviating Hypoxia. Cancer Cell (2014) 26(5):605–22. doi: 10.1016/j.ccell.2014.10.006 PMC4269830 25517747

[5] Danhier F Le Breton A Préat V. RGD-Based Strategies to Target Alpha(V) Beta(3) Integrin in Cancer Therapy and Diagnosis. Mol Pharm (2012) 9(11):2961–73. doi: 10.1021/mp3002733 22967287

[6] Beer AJ Haubner R Sarbia M Goebel M Luderschmidt S Grosu AL. Positron Emission Tomography Using [18F]Galacto-RGD Identifies the Level of Integrin Alpha(V)Beta3 Expression in Man. Clin Cancer Res (2006) 12(13):3942–9. doi: 10.1158/1078-0432.CCR-06-0266 16818691

[7] Yu YP Wang Q Liu YC Xie Y. Molecular Basis for the Targeted Binding of RGD-Containing Peptide to Integrin αvβ3. Biomaterials (2014) 35(5):1667–75. doi: 10.1016/j.biomaterials.2013.10.072 24268666

[8] Gao S Wu H Li W Zhao S Teng X Lu H. A Pilot Study Imaging Integrin αvβ3 With RGD PET/CT in Suspected Lung Cancer Patients. Eur J Nucl Med Mol Imaging (2015) 42(13):2029–37. doi: 10.1007/s00259-015-3119-1 26153145

[9] Li L Zhao W Sun X Liu N Zhou Y Luan X. (18)F-RGD PET/CT Imaging Reveals Characteristics of Angiogenesis in non-Small Cell Lung Cancer. Transl Lung Cancer Res (2020) 9(4):1324–32. doi: 10.21037/tlcr-20-187 PMC7481644 32953507

[10] Li L Ma L Shang D Liu Z Yu Q Wang S. Pretreatment PET/CT Imaging of Angiogenesis Based on (18)F-RGD Tracer Uptake may Predict Antiangiogenic Response. Eur J Nucl Med Mol Imaging (2019) 46(4):940–7. doi: 10.1007/s00259-018-4143-8 30187104

[11] Liu J Wu L Liu Z Seery S Li J Gao Z. (18)F-RGD PET/CT and Systemic Inflammatory Biomarkers Predict Outcomes of Patients With Advanced NSCLC Receiving Combined Antiangiogenic Treatment. Front Oncol (2021) 11:671912. doi: 10.3389/fonc.2021.671912 34150635 PMC8212050

[12] Noman MZ Desantis G Janji B Hasmim M Karray S Dessen P. PD-L1 is a Novel Direct Target of HIF-1α, and its Blockade Under Hypoxia Enhanced MDSC-Mediated T Cell Activation. J Exp Med (2014) 211(5):781–90. doi: 10.1084/jem.20131916 PMC4010891 24778419

[13] Chang YL Yang CY Lin MW Wu CT Yang PC. High Co-Expression of PD-L1 and HIF-1α Correlates With Tumour Necrosis in Pulmonary Pleomorphic Carcinoma. Eur J Cancer (2016) 60:125–35. doi: 10.1016/j.ejca.2016.03.012 27107327

[14] Chen J Jiang CC Jin L Zhang XD. Regulation of PD-L1: A Novel Role of Pro-Survival Signalling in Cancer. Ann Oncol (2016) 27(3):409–16. doi: 10.1093/annonc/mdv615 26681673

[15] Wan W Guo N Pan D Yu C Weng Y Luo S. First Experience of 18F-Alfatide in Lung Cancer Patients Using a New Lyophilized Kit for Rapid Radiofluorination. J Nucl Med (2013) 54(5):691–8. doi: 10.2967/jnumed.112.113563 PMC3683452 23554506

[16] Liu J Wang D Meng X Sun X Yuan S Yu J. 18F−Alfatide Positron Emission Tomography may Predict Anti−Angiogenic Responses. Oncol Rep (2018) 40(5):2896–905. doi: 10.3892/or.2018.6692 30226599

[17] Tran PN Sarkissian S Chao J Klempner SJ. PD-1 and PD-L1 as Emerging Therapeutic Targets in Gastric Cancer: Current Evidence. Gastrointest Cancer (2017) 7:1–11. doi: 10.2147/GICTT.S113525 28757801 PMC5533281

[18] Scheel AH Dietel M Heukamp LC Jöhrens K Kirchner T Reu S. Harmonized PD-L1 Immunohistochemistry for Pulmonary Squamous-Cell and Adenocarcinomas. Mod Pathol (2016) 29(10):1165–72. doi: 10.1038/modpathol.2016.117 27389313

[19] Grizzi F Castello A Qehajaj D Toschi L Rossi S Pistillo D. Independent Expression of Circulating and Tissue Levels of PD-L1: Correlation of Clusters With Tumor Metabolism and Outcome in Patients With non-Small Cell Lung Cancer. Cancer Immunol Immunother (2019) 68(9):1537–45. doi: 10.1007/s00262-019-02387-9 PMC11028209 31482306

[20] Li C Li C Zhi C Liang W Wang X Chen X. Clinical Significance of PD-L1 Expression in Serum-Derived Exosomes in NSCLC Patients. J Transl Med (2019) 17(1):355. doi: 10.1186/s12967-019-2101-2 31665020 PMC6820965

[21] Krock BL Skuli N Simon MC. Hypoxia-Induced Angiogenesis: Good and Evil. Genes Cancer (2011) 2(12):1117–33. doi: 10.1177/1947601911423654 PMC3411127 22866203

[22] Barsoum IB Smallwood CA Siemens DR Graham CH. A Mechanism of Hypoxia-Mediated Escape From Adaptive Immunity in Cancer Cells. Cancer Res (2014) 74(3):665–74. doi: 10.1158/0008-5472.CAN-13-0992 24336068

[23] You L Wu W Wang X Fang L Adam V Nepovimova E. The Role of Hypoxia-Inducible Factor 1 in Tumor Immune Evasion. Med Res Rev (2021) 41(3):1622–43. doi: 10.1002/med.21771 33305856

[24] Smith V Mukherjee D Lunj S Choudhury A Hoskin P West C. The Effect of Hypoxia on PD-L1 Expression in Bladder Cancer. BMC Cancer (2021) 21(1):1271. doi: 10.1186/s12885-021-09009-7 34819027 PMC8613983

[25] Shi Y Wu H Zhang M Ding L Meng F Fan X. Expression of the Epithelial-Mesenchymal Transition-Related Proteins and Their Clinical Significance in Lung Adenocarcinoma. Diagn Pathol (2013) 8:89. doi: 10.1186/1746-1596-8-89 23706092 PMC3671218

[26] Polyak K Weinberg RA. Transitions Between Epithelial and Mesenchymal States: Acquisition of Malignant and Stem Cell Traits. Nat Rev Cancer (2009) 9(4):265–73. doi: 10.1038/nrc2620 19262571

[27] Zuo J Wen J Lei M Wen M Li S Lv X. Hypoxia Promotes the Invasion and Metastasis of Laryngeal Cancer Cells *via* EMT. Med Oncol (2016) 33(2):15. doi: 10.1007/s12032-015-0716-6 26749588

[28] Higashi K Ueda Y Shimasaki M Ishigaki Y Nakamura Y Oguchi M. High FDG Uptake on PET is Associated With Negative Cell-to-Cell Adhesion Molecule E-Cadherin Expression in Lung Adenocarcinoma. Ann Nucl Med (2017) 31(8):590–5. doi: 10.1007/s12149-017-1187-y 28677069

[29] Togo M Yokobori T Shimizu K Handa T Kaira K Sano T. Diagnostic Value of (18)F-FDG-PET to Predict the Tumour Immune Status Defined by Tumoural PD-L1 and CD8(+)tumour-Infiltrating Lymphocytes in Oral Squamous Cell Carcinoma. Br J Cancer (2020) 122(11):1686–94. doi: 10.1038/s41416-020-0820-z PMC7250916 32238919

[30] Mu W Jiang L Shi Y Tunali I Gray JE Katsoulakis E. Non-Invasive Measurement of PD-L1 Status and Prediction of Immunotherapy Response Using Deep Learning of PET/CT Images. J Immunother Cancer (2021) 9(6):e002118. doi: 10.1136/jitc-2020-002118 34135101 PMC8211060

[31] Niemeijer AN Leung D Huisman MC Bahce I Hoekstra OS van Dongen G. Whole Body PD-1 and PD-L1 Positron Emission Tomography in Patients With non-Small-Cell Lung Cancer. Nat Commun (2018) 9(1):4664. doi: 10.1038/s41467-018-07131-y 30405135 PMC6220188

[32] Xing Y Chand G Liu C Cook GJR O'Doherty J Zhao L. Early Phase I Study of a (99m)Tc-Labeled Anti-Programmed Death Ligand-1 (PD-L1) Single-Domain Antibody in SPECT/CT Assessment of PD-L1 Expression in Non-Small Cell Lung Cancer. J Nucl Med (2019) 60(9):1213–20. doi: 10.2967/jnumed.118.224170 PMC6735283 30796165

